# Supplementary material for: Simultaneously Monitoring Whole Corneal Injury with Corneal Optical Density and Thickness in Patients Undergoing Cataract Surgery
Source: Diagnostics (Basel). 2021 Sep 7;11(9):1639. doi: 10.3390/diagnostics11091639 (PMC8471687; doi:10.3390/diagnostics11091639)
Supplement: Supplementary file 1 [file diagnostics-11-01639-s001.zip › diagnostics-1295448-supplementary.pdf]

Supplementary Materials

Supplementary Table S1. Preoperative quality assessment of Pentacam AxL® examination.

	Number (Eye)	Percent
OK	46	61.3%
AXL SNR Error	6	8.0%
Lid closure !	6	8.0%
Alignment (Z) Error	5	6.7%
Blinking Error !	5	6.7%
Unsteady Fixation	3	4.0%
AXL SNR Error Single Peak	2	2.7%
AXL Align. (XY) Error	1	1.3%
Data Gaps !	1	1.3%
Total	75	100.0%

Supplementary Table S2. Postoperative quality assessment of Pentacam AxL® examination.

	Number (Eye)	Percent
OK	50	66.7%
Lid closure !	8	10.7%
Alignment (Z) Error	4	5.3%
Unsteady Fixation	4	5.3%
Alignment (XY) Error	2	2.7%
Blinking Error !	2	2.7%
3D Model Deviation !	2	2.7%
AXL Align. (XY) Error	1	1.3%
Data Gaps !	1	1.3%
Blinking Error/Nose Shadow!	1	1.3%
Total	75	100.0%
